# Isolated Invasive Endomyocardial Cystic Echinococcosis Presenting with Heart Failure

**DOI:** 10.1155/2012/603087

**Published:** 2012-07-05

**Authors:** Suleyman Ercan, Vuslat Bosnak, Murat Yuce, Vedat Davutoglu, Fethi Yavuz

**Affiliations:** ^1^Department of Cardiology, School of Medicine, Gaziantep University, 27310 Gaziantep, Turkey; ^2^Department of Infectious Diseases, School of Medicine, Gaziantep University, 27310 Gaziantep, Turkey

## Abstract

Cardiac cystic *echinococcosis* is a rarely encountered parasitic infestation caused by *Echinococcus granulasus* larvae. Cystic *echinococcosis* hydatid composes 0.5–2% of all human cystic *echinococcosis* cases. Isolated cardiac involvement is very rare. Cardiac cystic *echinococcosis* hydatid generally accompanies another organ involvement, however, it might be isolated as in the present case and although rare and it can cause heart failure. We present a case of isolated apical cardiac cystic *echinococcosis* hydatid which leads to heart failure.

## 1. Introduction

Cardiac cystic *echinococcosis* is a rarely encountered parasitic infestation caused by *Echinococcus granulasus* larvae [1, 2]. Cardiac cystic *echinococcosis* composes 0.5–2% of all human cystic *echinococcosis* cases. The most commonly involved organs are liver (55–70%) and lung (18–35%) [3]. Cardiac cystic *echinococcosis* usually accompanies other organ involvement. Isolated cardiac involvement is very rare. We present a case of isolated apical cardiac cystic *echinococcosis* which leads to heart failure.

## 2. Case Presentation

**Figure 1:**
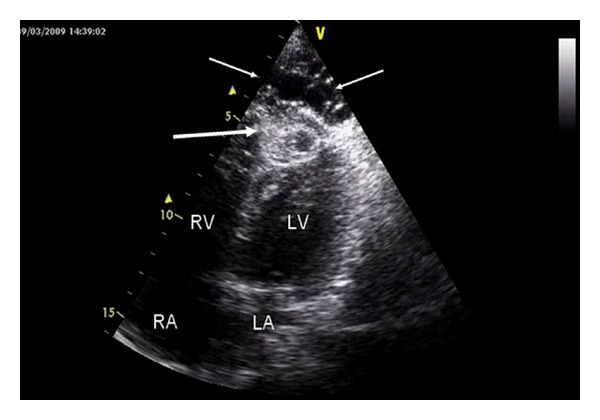
Apical four chamber view, revealing multiple hydatid cysts. Multiple small cysts surrounding and obliterating the apical region (small arrows). Relatively bigger cyst invasing the apicoseptal region (big arrow).

**Figure 2:**
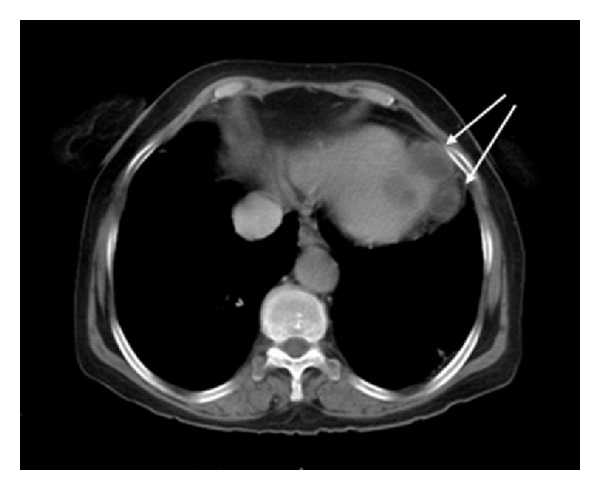
Computed tomography revealing multiple cystic *echinococcosis*. Multiple cysts surrounding apical region (arrows).

A 77-years-old female was referred to our clinic for evaluation of worsening heart failure. She had no history of international travel. During etiological evaluation of heart failure, echocardiography revealed an ejection fraction of 40% with a multilobular cystic structure localized to intramyocardial left ventricular apex (Figure 1). Computed tomography confirmed the diagnosis (Figure 2) and screening of other organ involvement including brain, lung, and liver were negative for cystic *echinococcosis*. Serum indirect hemagglutination assay test for *Echinococcus granulasus *was positive. Thus, diagnosis of isolated cardiac apical cystic *echinococcosis *  was confirmed. The patient was recommended surgery, however, patient refused the surgical operation. 

## 3. Discussion

Cystic *echinococcosis* is a parasitic infestation seen endemically in South America, South Europe, Africa, Turkey, Australia, New Zealand, and India due to *E. granulosus* [1, 2]. Larvae of *E. granulasus* are excreted in feces of definite hosts like cat, dog, and wolf. Humans are infected by the food contaminated with these larvae and become intermediate hosts [3].

Cyst hydatid mostly involves liver (55–70%) and lung (17–35%), however, cardiac involvement is rare (0.5–2%). Cardiac involvement are usually accompanied by other organ involvement [4, 5]. Cardiac cystic *echinococcosis* involves most commonly left ventricle (75–55%), right ventricle (15–18%), interventricular septum (5–9%), right atrium (3-4%), and interatrial septum (2%). Less commonly it involves pericardium and pulmonary artery. Cardiac cystic *echinococcosis* most commonly involves left ventricular wall possibly due to increased coronary blood flow [6]. 

Cardiac cystic *echinococcosis* is usually asymptomatic. Symptoms vary according to localization and dimensions of the cyst. Patients usually present with three main symtoms: dyspnea, chest pain, and palpitations. Other rare symptoms include fever, cough, hemoptysis, syncope, and sudden death. Our patient was suffering from dyspnea due to pulmonary edema.

Although cystic *echinococcosis* grows usually very slowly in the myocardium and may remain asymptomatic for a long period it can also show rapid progression [7]. Early diagnosis of cardiac cystic *echinococcosis* is rather difficult beacuse of long interval between beginning of the infestation and development of symptoms and besides symptoms are nonspecific [8].

Diagnosis of cardiac cystic *echinococcosis* is easier in the presence of other organ involvement like liver and lung, but it is difficult in isolated cardiac involvement. Although CT and MRI give detailed information about the cyst and its localization, echocardiography is the best noninvasive method for diagnosis of cardiac cystic *echinococcosis* [9].

Although the course of cardiac cystic *echinococcosis* is usually asymptomatic, it may cause life-threatening complications including sudden death, anaphylactic shock, peripheral pulmonary emboli and cerebral emboli, acute coronary syndrome, heart failure, and arrhythmia. Therefore, cardiac cystic *echinococcosis* should be treated by surgical intervention even they are asymptomatic.

In conclusion, cardiac cystic *echinococcosis* is generally accompanies other organ involvement, however, it might be isolated as in the present case and although rare, and it can cause heart failure.
